# Randomly Distributed Fabry-Pérot-type Metal Nanowire Resonators and Their Lasing Action

**DOI:** 10.1038/srep24898

**Published:** 2016-04-22

**Authors:** Kyungmok Kwon, Youngho Jung, Minkyung Kim, Jaeho Shim, Kyoungsik Yu

**Affiliations:** 1 School of Electrical Engineering, KAIST, 291 Daehak-ro, Yuseong-gu, Daejeon, 34141, Korea

## Abstract

Optical feedback mechanisms are often obtained from well-defined resonator structures fabricated by top-down processes. Here, we demonstrate that two-dimensional networks of metallic nanowires dispersed on the semiconductor slab can provide strong in-plane optical feedback and, thus, form randomly-distributed Fabry-Pérot-type resonators that can achieve multi- or single-mode lasing action in the near infrared wavelengths. Albeit with their subwavelength-scale cross-sections and uncontrolled inter-nanowire distances, a cluster of nearly parallel metal nanowires acts as an effective *in-situ* reflector for the semiconductor-metal slab waveguide modes for coherent optical feedback in the lateral direction. Fabry-Pérot type resonance can be readily developed by a pair of such clusters coincidentally formed in the solution-processed random nanowire network. Our low-cost and large-area approach for opportunistic random cavity formation would open a new pathway for integrated planar light sources for low-coherence imaging and sensing applications.

Conventional semiconductor-based lasers with well-defined optical resonator structures are typically obtained from top-down or *subtractive* fabrication processes, including multiple growth, lithography and/or etching steps, to unambiguously define the laser cavity boundary by refractive index contrasts. Although such optical resonator structures^1^ may offer excellent performances with well-defined resonance wavelengths and high quality factors, they require additional fabrication processing cost and tight precision tolerance limit.

In this paper, we propose an *additive* and facile fabrication approach to opportunistically form planar resonators with random deposition of silver nanowires on a semiconductor thin film without relying on any lithographic patterning and etching processes. We take advantage of subwavelength-scale modification of the surface refractive index profile with simple solution-based drop-casting of passive metal nanowires to establish a feedback mechanism within the flat optical gain material. Rather than randomly placing the optical gain media as in many random laser cases^2^,^3^,^4^, we introduce randomly-distributed optical reflectors^5^,^6^ on the continuous III-V compound semiconductor layer to facilitate in-plane optical feedback within the thin semiconductor slab layer by opportunistic cavity formation, as schematically shown in Fig. 1a. Although the reflectors and scatterers are placed on the semiconductor surface without any predefined patterns, the randomness of cavities supports multiple emission peaks over the broad spectrum and angular distribution. Such properties can be exploited in a number of applications including speckle-free illumination and imaging^7^,^8^. Two-dimensional random networks of metal nanowires have been extensively studied for transparent and flexible conducting electrodes^9^,^10^,^11^, but their applications on in-plane cavity formation for lateral distributed optical feedback have never been studied in detail.

An important question on implementing this idea is whether simple topographical and/or refractive index profile modification of the semiconductor thin film surface, such as the deposition of nanowires and nanoparticles with subwavelength-scale cross-sections, can provide enough in-plane reflection to induce sufficient optical feedback for cavity formation. One-dimensional metal nanowires are an excellent candidate in this context since they strongly interact with the electromagnetic waves and thus have been studied for plasmonic resonators^12^,^13^,^14^ and waveguides^15^,^16^,^17^.

## Results and Discussion

**Figure 1:**
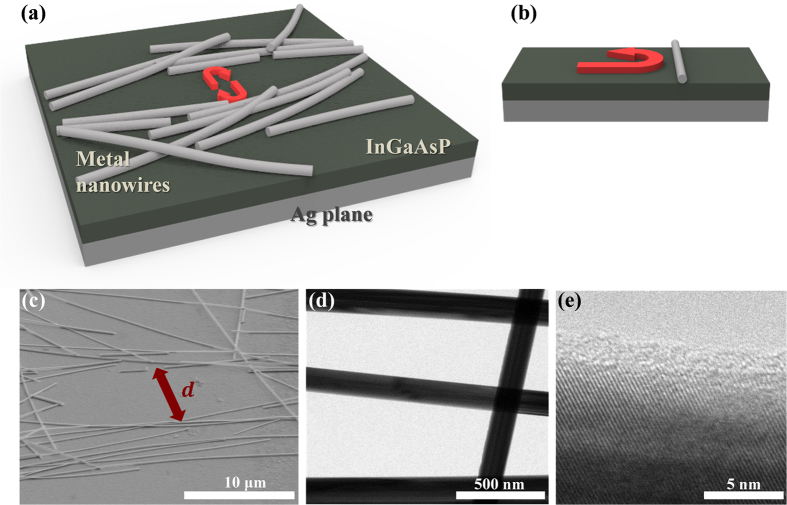
(**a**) A schematic illustration of the proposed thin film lateral optical resonator defined by randomly distributed multiple metal nanowires. (**b**) Simplified view of a subwavelength-scale metal nanowire reflector. Metal nanowires with subwavelength-scale cross-section dimensions can efficiently reflect the guided electromagnetic waves with the matched polarization and mode profile. (**c**) A scanning electron micrograph of an InGaAsP thin film resonator/laser with a drop-casted silver nanowire network and a metal substrate (52° tilted view). The cavity length or the distance between the nanowire reflectors is *d.* (**d**,**e**) TEM images of single-crystalline silver nanowires with a nominal diameter of ~120 nm. The nanowire surface is coated with a thin (<2 nm) cladding layer.

Figure 1b schematically illustrates light reflection from the elementary, subwavelength-scale perturbation of the refractive index profile on the dielectric slab surface. To facilitate stronger interaction of electromagnetic waves with such minute perturbation, we employ an additional metal bottom plane for tight electromagnetic field confinement. In Fig. 1b, the refractive index perturbation on the semiconductor film is created by a lossy metal nanowire with a large negative permittivity. Although the index profile near the surface can be modified by corrugating the slab surface or placing a dielectric nanowire similar to the surface grating for Bragg reflection, such a single dielectric grating element has only <20% reflectivity for the guided modes at the wavelength range of our interest (Fig. S1 in Supporting Information). Therefore, well-coordinated multiple dielectric grating elements with large footprints and accurate fabrication processes are therefore necessary to provide sufficient reflection for strong optical feedback. In contrast, a single metal nanowire with the same cross-sectional area can support >50% reflectivity in a wide range of wavelengths when the slab waveguide mode is normally incident on the nanowire axis.

Figure 1a shows a schematic example of the proposed semiconductor thin film optical resonator and laser opportunistically defined by random metal nanowire networks on the semiconductor-metal slab without lithographic patterning and etching processes. If metal nanowires efficiently reflect the light propagation in the dielectric slab region, the guided slab modes can be trapped within the opening area of the nanowire network and start oscillating between the nanowire clusters, as illustrated by the red arrows in Fig. 1a. When the dielectric slab layer (thin film) is made of a semiconductor material with a sufficient optical gain that can overcome the propagation and reflection losses in the cavity, lasing action can be achieved from this optical feedback mechanism. Multiple metal nanowires placed closely with each other can reinforce the reflectivity and provide even stronger optical confinements between the nanowire clusters. This effect occurs even when the nanowires are not perfectly aligned with each other. Figure 1c shows an example of a randomly-distributed two-dimensional silver nanowire network for random formation of the optical laser cavities. The lateral Fabry-Pérot-type cavity length, *d*, is roughly defined by the distance between the nearly-parallel metal nanowire clusters, and it is approximately 10 μm in this particular example. As shown in Fig. 1d, the nominal diameter of the nanowires (~120 nm) is much smaller than the resonant wavelength as well as the overall cavity length scale. Since the guided modes for highly multimode dielectric slab waveguides with large core thicknesses do not efficiently interact with such subwavelength-scale index profile perturbation near the surface, we restrict our discussion to the thin dielectric semiconductor slabs (*n*_*slab*_*h* < *λ,* where *n*_*slab*_, *h*, and *λ* represent the slab’s refractive index, the slab thickness, and the operation wavelength, respectively.) supporting only a few fundamental guided modes, such as transverse-electric TE_0_ and TE_1_, for the near-infrared wavelength ranges. More detailed modal analysis for the slab waveguide geometry can be found in Fig. S3.

We first consider the reflection of the slab modes from a single silver nanowire (120 nm diameter, as observed in Fig. 1d) on a symmetric semiconductor slab suspended in air (*n*_*air*_ = 1, *n*_*slab*_ = 3.4) as shown in Fig. S2. To estimate the maximum amount of reflection from the metal nanowire reflector, its orientation is assumed to be perpendicular to the wave propagation direction. According to our simulations based on the two-dimensional finite-difference time-domain (FDTD) method, the maximum achievable reflectivity is less than only 10% for the slab thickness of *h* = 350 nm when the operation wavelength is around 1450 nm. Although the reflection may slightly increase as the dielectric slab thickness decreases, this level of reflectance and optical feedback is not sufficient to offer strong in-plane optical resonances for lasing at typical optical gain values for compound semiconductor materials.

**Figure 2:**
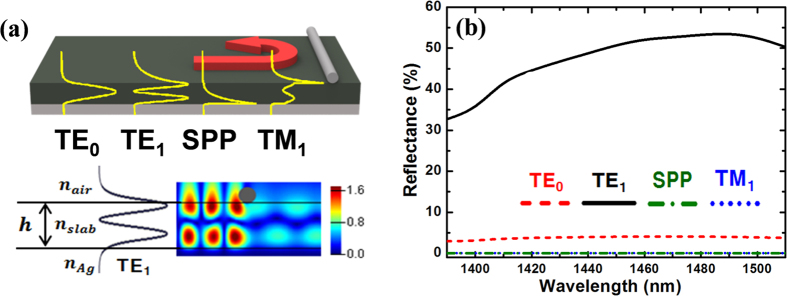
(**a**) Mode profile of the guided modes (TE_0_, TE_1_, SPP, TM_1_) and calculated electric field intensity distribution when the TE_1_ mode is incident on the silver nanowire reflector. (**b**) Calculated reflectance of the guided modes (TE_0_, TE_1_, SPP, TM_1_) from a single silver nanowire on an asymmetric slab waveguide with a silver substrate. The calculated electric field intensity distribution for the incident TE_1_ mode shows stronger reflection and weaker transmission from the nanowire reflectors. The maximum reflectance of 54% is obtained.

To provide stronger reflection from the metal nanowire placed on the slab-air interface, the guided mode profile should have a better spatial overlap with the nanowire reflector. From this rationale, we employ an asymmetric slab geometry with a metal (silver) substrate to maximize the evanescent field intensity near the dielectric slab surface, as schematically described in Fig. 2a. The electric field intensities near the semiconductor slab surface become non-negligible, and the reflection from a single silver nanowire can be significantly increased to more than 50% for the transverse electric TE_1_ mode (black curve in Fig. 2b). Such large reflectance is observed for a broad wavelength range between 1450 nm and 1500 nm. We also find that the TE-polarized modes (electric field parallel to the nanowires) show significantly higher reflectance than the transverse magnetic (TM) modes (magnetic field parallel to the nanowires) because the metal nanowire orientation matches with the electric field oscillation direction. In particular, the TE_1_ mode has much stronger reflection than the other modes because of its larger spatial overlap with the nanowire on the surface. The electric field distributions of all four guided modes for the asymmetric slab waveguide (TE_0_, TE_1_, TM_1_, and surface plasmon polariton (SPP) mode for the slab thickness of *h* = 350 nm) are plotted in Fig. 2a.

**Figure 3:**
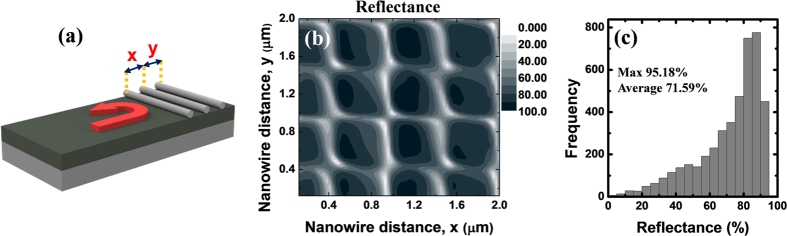
(**a**) A schematic view of a triple-metal-nanowire reflector. (**b**) Simulated reflectance from three parallel nanowires at 1450 nm. The dark areas represent the high reflection condition. (**c**) Histogram of the reflectance values in (**b**). The inter-nanowire distances (x and y) were varied from 120 nm to 2 μm, and 4356 cases were examined in total.

In general, it is possible to obtain even higher reflection with multiple reflectors. As an example, Fig. 3a–c present the TE_1_ mode reflectance from three parallel silver nanowires with various inter-nanowire distances at an operation wavelength of 1450 nm. To figure out the range of reflectivity values from the randomly-distributed metal nanowires, the reflectance in accordance with the distance between the nanowires was calculated. In most cases, large reflectance (>80%) can be obtained with just three metal nanowires. This implies that, albeit due to their randomness, metal nanowire clusters have the potential to provide sufficient reflection for the random formation of in-plane resonators in the semiconductor thin film. Meanwhile, the maximum reflectance from three dielectric grating elements (surface corrugations) is only ~70% as shown in Fig. S4a–c, and the average reflectance is below 50%. Although the maximum achievable reflectance from many dielectric grating elements can approach the unity, the metal nanowire clusters can actually provide higher reflectance values when only a few elements are involved.

If the slab mode propagation is trapped between two metal nanowire cluster reflectors with the effective reflectance value of *R*, the distributed round-trip loss for the nanowire-defined Fabry-Pérot type resonator is given by *α*_*p*_ − (1*/d*) ln *R*, where *α*_*p*_ is the propagation loss of the semiconductor-metal slab waveguide and *d* is the effective cavity length. The calculated distributed losses for the TE_1_ mode demonstrate the possibility of lasing from the nanowire-defined resonant structures (Fig. S5). Although the metal substrate layer introduces non-negligible propagation losses from its ohmic dissipation (*α*_*p*_ ~ 200 cm^−1^ at low temperature (77 K) was estimated by a numerical method), the total round trip loss for the metal nanowire-defined cavity is much smaller than the symmetric dielectric slab waveguides because of the reduced reflection losses (larger *R*). The effect of the increased reflection from the metal nanowires becomes more pronounced when the cavity dimensions (*d*) and the contribution of the propagation loss over the total cavity loss (*α*_*p*_*d*) become smaller. When multiple silver nanowires form a cluster and reflect the normally incident guided slab mode at each side of the Fabry-Pérot resonator with a maximum reflection of >95%, the distributed round-trip loss becomes as low as ~200 cm^−1^. Considering the mode confinement factors and the overall distributed losses at the same time, we expect that the lasing gain threshold can be as small as ~300 cm^−1^ (Fig. S6), which is easily attainable at low temperature for bulk InGaAsP materials^18^. With a few dielectric surface corrugations, however, it is difficult to reach the lasing condition because of too little optical feedback as indicated in Fig. S6. We also investigate the angular dependence of the reflectance from two non-parallel metal nanowires placed on the dielectric-metal slab (Fig. S7). Although the reflectance from the nanowires varies with the relative angle between them, metal nanowires with moderate angle deviation can still maintain high reflectance.

To verify our theoretical analysis, we fabricated a planar semiconductor slab (epitaxial InGaAsP material with the optical gain at near-infrared wavelengths around of 1450 nm) with a silver bottom layer. The semiconductor slab thickness was chosen as *h* = 350 nm to obtain strong reflections within the gain bandwidth of the semiconductor material as well as to guide only a few waveguide modes in the wavelength range of interest (see Fig. S3 for details). Single-crystalline silver nanowires with a nominal diameter of ~120 nm (Fig. 1d,e) were drop-casted on the semiconductor slab surface, and the randomly-distributed nanowires formed two-dimensional disordered networks, as shown in Fig. 1c. More details about the sample fabrication are described in the Experimental Section and Fig. S8.

**Figure 4:**
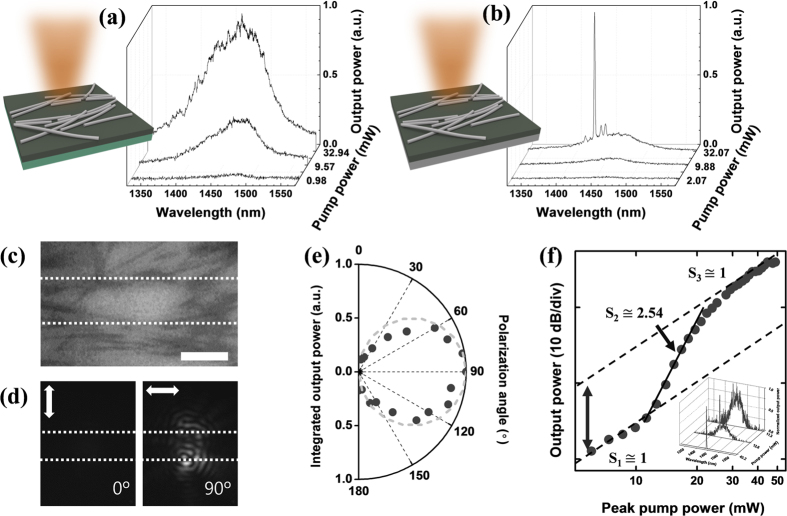
Optical characterization results. (**a**) Photoluminescence spectra from the InGaAsP epitaxial layer with the InP substrate (green). Low index contrast between the InGaAsP layer and the InP substrate hinders strong interaction between the guided mode and the silver nanowires. This experiment result together with Fig. 2 addresses the role of the metal substrate in formation of strong optical feedback. (**b**) Photoluminescence spectra from the InGaAsP epitaxial layer on the silver substrate (gray). The emission spectra evolution indicates multimode resonances for this simple geometry. (**c**) Optical microscope image of the measured resonator area. The scale bar represents 10 μm. (**d**) Infrared images with two orthogonal linear polarizer angles. The approximate locations of silver nanowire reflectors are shown with the dashed lines. (**e**) Integrated optical output power with respect to the polarization angle. The dashed circle represents the ideal linearly-polarized emission following the sinusoidal variation with respect to the polarization angle. (**f**) The relationship between the input and output optical power for the single-mode lasing case (inset of (**f**)) with the fitting lines for the spontaneous (S_1_), amplified spontaneous (S_2_), and stimulated emission regions (S_3_).

The optical characterization was performed by pumping the sample with 1064 nm laser pulses at various locations from the surface normal direction. Additional details on the measurements are provided in the Experimental Section. Figure 4 shows the photoluminescence and lasing characteristics of a nanowire-defined randomly distributed resonator structure at low temperature (77 K). At first, we investigated the effect of metal nanowire networks deposited on the compound semiconductor slab without the bottom metal plane as shown in Fig. 4a. Similarly with Fig. S2, the optical feedback was not sufficient to sustain a lasing condition in this case, and the photoluminescence emission spectra resemble the case without the metal nanowires. This result also proves that the resonance and lasing phenomena observed in Fig. 4b do not come only from the surface plasmon resonances of silver nanowires themselves, but also from the interaction between the nanowires and the bottom metal plane. The presence of the bottom metal plane and its interplay with the metal nanowires play important roles in confining the electromagnetic waves in the active region. After replacing the dielectric substrate with the silver bottom plane, we measured photoluminescence spectra with (Fig. 4b) and without nanowires (Fig. S9). The lasing operation was only observed when the metal nanowires locally form a nearly-parallel Fabry-Pérot-like cavity (see Supplementary Video in Supporting Information).

In the randomly distributed Fabry-Pérot resonator with the bottom silver plane, the excitation and lasing of the TE-polarized waveguide mode is confirmed by polarization-resolved measurements of scattered emission in Fig. 4d,e. Most emission collected from the surface normal direction is linearly polarized along the nanowire orientation (horizontal direction in Fig. 4d), which confirms that the dominant and lasing mode is indeed TE-polarized. The SPP resonant mode might exist along the nanowire^19^, but its relative output intensity is estimated to be much weaker than the TE mode. When the linear polarizer is configured perpendicular to the nanowires’ major axes, the out-of-plane laser output power obtained by an objective lens is negligible. The appearance of the fringe patterns above the lasing threshold in Fig. 4d indicates coherent emission from the nanowire-defined resonator.

Since the metal nanowires are randomly distributed by drop casting and, thus, are not perfectly aligned with each other, multiple resonant paths might exist between the metal nanowire cluster reflectors. When the optical feedback is strong enough to satisfy the lasing condition, optical resonance and lasing action can also be observed between non-parallel nanowire clusters as shown in Fig. S12. An example of a multimode lasing spectrum is shown in Fig. 4b. Similarly with the conventional random laser examples, we can selectively excite single or multiple cavity modes by varying the location, size, and intensity of the pump beam. To further investigate the lasing action more carefully, we intentionally select an optical pumping condition in which only one lasing peak is developed with respect to the input pump power. The evolution of the single-mode laser emission spectra and the integrated output power are shown in Fig. 4f, respectively. Because of the relatively short cavity length of *d* ~ 10 μm, the free spectral range of the resonator near the lasing wavelength (~21 nm) becomes comparable to the optical gain bandwidth of the InGaAsP semiconductor material, which allows the single-mode lasing action from the randomly-formed semiconductor cavity. The lasing wavelength (~1450 nm) coincides with the spectral range that shows high reflectivity from the metal nanowire reflector, as described in Figs 2 and 3. Large propagation losses from the bottom metal layer and low cavity quality factors result in relatively large threshold pump powers, which were typically greater than 10 mW in our measurements. Further improvement in the lasing threshold could be achieved by using high-quality metal substrates^20^ as well as semiconductor materials with larger optical gains^21^. As indicated in Fig. 4f, the S-shaped light-light curve in the log-log scale demonstrates the typical single-mode laser behavior. Both the spontaneous and stimulated regions have a slope (S_1_ and S_3_) of ~1 in this plot. A relatively small change of the emission efficiency before and after the lasing threshold (about a factor of 10 as noted by an arrow in Fig. 4f) indicates small nonradiative surface recombination rates and minimal damage to the active optical materials resulting from etchless definition of the optical resonator structures with a simple solution-processed metal nanowire deposition.

Due to the band filling effect of the semiconductor materials, the cavity resonance moves quickly toward the shorter wavelengths with the increasing pump powers and carrier densities (Fig. S10). Continuous blue shifts, even at high pump powers, demonstrate efficient heat dissipation through the metal layer. The measured linewidth of ~3 nm near the lasing threshold is comparable to the calculated quality factors in Fig. S5.

## Conclusion

In summary, we experimentally demonstrated facile and opportunistic formation of thin film planar Fabry-Pérot cavities using randomly-placed two-dimensional metal nanowire networks on the semiconductor slab surface. The metal nanowires with subwavelength-scale cross-sections were randomly deposited by a simple solution-based drop-casting process and, therefore, offer a new route to obtain in-plane optical resonances without additional fabrication processes, such as lithographic patterning and etching, typically required for conventional optical resonators and lasers. Since the transverse electric mode reflection increases with the density and population of metal nanowires, the Fabry-Pérot resonators formed by the suggested method can achieve sufficient quality factors for lasing action. Although our demonstration mainly focused on the bulk compound semiconductor materials and the near-infrared wavelengths, the same principle can be applied to other material/wavelength combinations, such as semiconductor quantum wells with larger optical gain levels at the visible wavelengths. When further combined with advanced nanowire manipulation^22^,^23^,^24^ and deposition^25^,^26^ as well as nanowire-based transparent conductive electrode^27^,^28^ structures, the proposed scheme can provide an efficient pathway for both current injection and photon confinement in various thin film optoelectronic devices and low-coherence light sources^29^.

## Methods

### Fabrication

Compound semiconductor layers (30 nm InGaAs, 8 nm InP, 310 nm InGaAsP, 8 nm InP) were grown on an InP substrate by metal-organic vapor phase epitaxy, and additional metal layers (10 nm Ti, 300 nm Ag, 30 nm Pt, 2000 nm Ag) were electron-beam evaporated. It was bonded to a silver-evaporated silicon wafer with BiSn solder foil for two minutes at 170 degrees Celsius using a flip-chip bonder (FC-150, Karl Suss). The low bonding temperature and short bonding time were used to minimize metal diffusion. The InP substrate was then removed by mechanical grinding and chemical etching. In the final step, silver nanowires (Sigma-Aldrich) dispersed in isopropanol were drop-casted by a micro-pipette, and the planar optical resonator structures were randomly formed in the two-dimensional metal nanowire network. The length and diameter of silver nanowires were 30 ± 10 μm and 120 ± 10 nm, respectively. To reduce the optical loss and enhance the nanowire-light interaction, single crystalline nanowires with a thin dielectric cladding layer (~1.5 nm) were used (Fig. 1e). The optical effects of the cladding layer are examined as in Fig. S12a, and the reflectivity degradation from the cladding layer with a thickness of <2 nm was found to be minimal. We also investigated the effects of nanowire diameter on reflectance in Fig. S12b.

### Optical characterization

We used a micro-photoluminescence setup to measure the emission spectra from various metal nanowire configurations. The sample was placed in a cryostat chamber cooled by liquid nitrogen (77 K) to enhance the material optical gain and to reduce nonradiative recombination and metal’s optical absorption. A 1064 nm pulsed diode laser optically pumped the sample through a microscope objective with the numerical aperture of 0.5 from the surface normal direction. A pump pulse width of 10 ns and a period of 5000 ns were chosen to minimize any thermal effects. The effect of nanowire orientation to the pump beam absorption is explained in Fig. S13. Vertically scattered light emission from the sample was collected through the same objective and analyzed using an infrared spectrometer with a cooled detector array (iDus InGaAs 1.7, Andor). For polarization-resolved near-field radiation images, the same setup was used again, but, instead of the spectrometer, a two-dimensional infrared imaging detector (XEVA-FPA-1.7–640, Xenics) was placed at the image plane of the objective. The polarized images were captured by inserting a broadband linear polarizer in front of the detector.

### Numerical Simulations

We assume that the real part of the silver permittivity does not change much with temperature, and use the known room-temperature values^30^ for the simulations. For the imaginary part of the silver permittivity, we used half of the room-temperature values because the imaginary part of the metal permittivity decreases with temperature owing to reduced free-electron phonon scattering. A similar approach has been used in the literature^31^,^32^. The guided optical modes for the semiconductor slab layer with and without the bottom metal layer were analyzed by finite difference eigenmode solver, and their optical properties, such as propagation loss (*α*_*p*_), confinement factor, are verified again with two-dimensional FDTD simulations. The reflectance values for various scenarios were obtained by two-dimensional FDTD simulations.

## Additional Information

**How to cite this article**: Kwon, K. *et al.* Randomly Distributed Fabry-Pérot-type Metal Nanowire Resonators and Their Lasing Action. *Sci. Rep.* **6**, 24898; doi: 10.1038/srep24898 (2016).

## Supplementary Material

Supplementary Information

Supplementary Video
