# Supplementary material for: Randomly Distributed Fabry-Pérot-type Metal Nanowire Resonators and Their Lasing Action
Source: Sci Rep. 2016 Apr 22;6:24898. doi: 10.1038/srep24898 (PMC4840363; doi:10.1038/srep24898)
Supplement: Supplementary Information [file srep24898-s1.doc]

Supplementary Information

**Randomly Distributed Fabry-Pérot-type Metal Nanowire Resonators and Their Lasing Action**

*Kyungmok Kwon, Youngho Jung, Minkyung Kim, Jaeho Shim, and Kyoungsik Yu**

*School of Electrical Engineering, KAIST*

*291 Daehak-ro, Yuseong-gu, Daejeon, 34141, Korea*

**email: ksyu@kaist.edu*

**Figure S1.** (a) Calculated reflectance of the guided modes (TE_0_, TE_1_, TM_0_, TM_1_) from subwavelength-scale dielectric index perturbation (a surface corrugation grating element, 120 nm by 120 nm) on a symmetric dielectric slab waveguide suspended in air. The maximum reflectance is below 8% over the wavelength range of our interest. (b) Calculated reflectance of the guided slab modes (TE_0_, TE_1_, SPP, TM_1_) from a surface corrugation grating element on an asymmetric slab waveguide with a silver substrate. The maximum reflectance is better than (a), but worse than a metal nanowire on the asymmetric slab waveguide with the silver substrate shown in Figure 2.

**Figure S2.** Calculated reflectance of the guided modes (TE_0_, TE_1_, TM_0_, TM_1_) from a single silver nanowire (120 nm diameter) on a symmetric dielectric slab waveguide suspended in air (*h*=350 nm). The inset also shows the refractive index profile as well as the calculated electric field intensity distribution when the TE_1_ mode is incident on the silver nanowire reflector.

**Figure S3.** Dispersion relationship for the (a) symmetric and (b) asymmetric slab waveguide with the dielectric core (InGaAsP material) thickness of *h*=350 nm. Only TE_0_, TE_1_, TM_0_, TM_1_ (TE_0_, TE_1_, SPP, TM_1_) modes are supported in the symmetric (asymmetric) slab within the material gain bandwidth indicated in gray.

**Figure S4.** (a) A schematic view of a triple-dielectric-grating reflector. (b) Simulated reflectance from three dielectric grating elements at 1450 nm. The average reflectance is significantly inferior when compared with the metal nanowire case. (c) Histogram of the reflectance values in (b).

**Figure S5.** (a) A schematic view of a triple-metal-nanowire reflector with the inter-nanowire spacing of x and y. (b) The distribution of the quality factors for Fabry-Pérot-like cavities with two parallel triple-metal-nanowire reflectors shown in (a). The reflectivity of the triple-metal-nanowire reflector, *R*, is obtained from Figure 3a. The wavelength and the cavity length are assumed to be *λ*=1450 nm and *d*=10 μm, respectively.

The total cavity quality factor, *Q*, can be obtained by


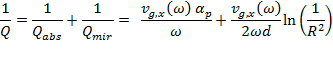


where *ω* is the resonance frequency, *v_g_(ω)* the group velocity of the guided mode, *α_p_* the propagation absorption loss, *d* the cavity length, and *R* the average reflectivity from the metal nanowire clusters.[1] Since the proposed optical resonator has an absorption quality factor, *Q_abs_*, of over 1100 and a mirror reflection loss quality factor, *Q_mir_*, of a few hundred, the reflection loss is dominant. This implies that the density of metal nanowires plays an important role in resonator performance.

**Figure S6.** (a) A schematic view of a triple-metal-nanowire reflector. (b) Calculated lasing threshold gain level in the unit of cm^-1^ with respect to the inter-nanowire distances, x and y. It is highly probable that low gain threshold values of <350 cm^-1^ can be achieved with nanowire cluster reflectors with more than three silver nanowires. The lasing gain threshold is given by *g_th_*= *α_tot_*/*Γ*=*α_p_*-(1*/d*)ln*R* where *Γ* is the confinement factor and *α_tot_* is the distributed total loss. The confinement factor for the TE_1_ mode, *Γ* (0.855 at 1450 nm), was obtained from the ratio of the electromagnetic power inside the waveguide region over the total power using the eigenmode solver. (c) A schematic view of a triple-dielectric-grating reflector. (d) Calculated lasing threshold gain level with respect to the grating element distances, x and y. The triple-dielectric-grating reflector requires much higher optical gain values to achieve the lasing condition.

**Figure S7.** Angular dependence of the reflectance from two non-parallel metal nanowires placed on the dielectric-metal slab. Although the reflectance from the nanowires increases with the relative angle between them, two non-parallel metal nanowires can still maintain high reflection when the angle, *θ*, is moderate. Although not shown here, this trend holds for three or more metal nanowires

**Figure S8.** Schematic diagram of the sample preparation process. (a) Epitaxial layer growth and metal deposition. (b) Deposition of a BiSn foil and a silver bonding layer on the supporting silicon wafer for (c) thermocompressive metal-metal bonding. (d) Drop-casting of silver nanowires after removing the InP substrate.

**Figure S9.** PL spectra from the asymmetric slab waveguide (InGaAsP epitaxial layer) with the silver substrate. Broad spontaneous emission spectrum was observed due to the absence of optical feedback.

**Figure S10.** Peak emission wavelength as a function of the optical pumping power. Due to the band filling effect in the semiconductor material, the peak wavelength moves toward the shorter wavelengths as the pump power increases.

**Figure S11.** (a) A scanning electron micrograph of an InGaAsP thin film cavity with non-parallel silver nanowires. (b) An infrared image of the lasing cavity. (c) Photoluminescence spectra evolution, and (d) its output power as a function of the optical pump power.

**Figure S12.** (a) Reflectance degradation of the TE_1_ mode due to the additional dielectric cladding layer outside the metal nanowire. (b) The TE_1_ mode reflectance with various nanowire diameters.

The polymer cladding material outside the metal nanowires, such as PVP (polyvinyl pyrrolidone), can degrade the metal nanowire reflectivity. To investigate its effect quantitatively, the metal nanowire with a thin dielectric outer shell (refractive index of 1.5) was used for computer simulations. If the outer cladding thickness is less than a few nanometers, the reflectance degradation remains within just a few percent. According to (b), metal nanowires with larger diameters can provide stronger reflection since they have a spatial overlap with the TE_1_ mode. The nanowire diameters used in our experiments (~120 nm) show decent reflectance values over the wavelength range of our interest.

**Figure S13.** Calculated absorption from the semiconductor slab with respect to the incident pump beam polarization and the presence of a silver nanowire and a silver substrate (gray circle and gray rectangular box). (a) Absorptance without a silver nanowire was calculated to be ~78%. Absorptance with a silver nanowire for (b) TM- and (c) TE-polarized pump beam were 72% and 80%, respectively. When the pump beam polarization matches with nanowire orientation, absorption at the semiconductor layer decreases (from ~78% to ~72%) due to reflection of the pump beam. However, when the pump beam polarization is perpendicular to the nanowire axis, the overall absorption can be slightly enhanced due to the excitation of localized surface plasmons at the metal-semiconductor interface.

Reference

1. Chang, S.-W.; Lin, T.-R.; Chuang, S. L., Theory of plasmonic Fabry-Perot nanolasers. *Opt. Express* **2010,** *18* (14), 15039-15053.
